# Effects of Nutritional Status and Foods Consumed on Inflammation and Disease Activity in Patients with Rheumatoid Arthritis

**DOI:** 10.3390/medicina60081197

**Published:** 2024-07-24

**Authors:** Bilal Uysal, Nilay Sahin, Hayrettin Kara

**Affiliations:** 1 Faculty of Medicine, Balikesir University, 10145 Balıkesir, Türkiye; dincernilay@yahoo.com; 2 Health Practice and Research Hospital, Balikesir University, 10145 Balıkesir, Türkiye; dythayrettinkara@gmail.com

**Keywords:** rheumatoid arthritis, nutrition, inflammation, disease activity

## Abstract

*Background and Objectives:* This study investigated the impact of nutritional status and foods consumed on inflammation and disease activity in patients with rheumatoid arthritis (RA). *Materials and Methods:* We designed a cross-sectional observational study, involving 110 patients diagnosed with RA. The patients included were between 18 and 75 years old, diagnosed with rheumatoid arthritis two years ago or earlier, with stable treatment for the last 8 weeks. Data on anthropometric parameters, body mass composition, nutritional status, individual food consumption records, inflammation, disease activity, quality of life, clinical, and laboratory parameters were collected for each study participant. The evaluation parameters of the patients were the simple disease activity index (SDAI), clinical disease activity index (CDAI), systemic immune-inflammation index (SII) and individual food consumption records. A bioimpedance device and measuring tape were used to take body composition and anthropometric measurements of the patients. *Results:* According to the body mass index, waist circumference and waist-to-height ratio, in our study, we found that 60% of the patients were obese, 80% were at a very high health risk, and approximately 91% were in need of nutritional treatment. There was a significant negative correlation between the dietary intake of total energy, total fat, omega 3, calcium, zinc, cobalamin and the disease activity (SDAI, CDAI). There was a significant negative correlation between polyunsaturated fatty acids, omega 3, carotene, vitamin E, selenium and the SII. Additionally, there was a positive correlation between omega 6 and the SII, SDAI, CDAI (*p* < 0.05). *Conclusions:* The results of this study show that the foods consumed in the nutrition of RA patients may have effects on their inflammation and disease activity.

## 1. Introduction

Rheumatoid arthritis (RA) is a chronic, progressive, inflammatory disease characterized by symmetrical polyarthritis and accompanied by systemic involvement [1,2]. RA affects both the physical and mental health of patients, reducing their quality of life and decreasing their capacity to work. Therefore, it creates serious economic costs, both individually and socially [3,4].

RA is an autoimmune disease of uncertain etiology, affecting ~0.5–1% of the population, characterized by systemic inflammation [3]. It has been reported that many combinations of genetic and environmental risk factors in the etiopathogenesis of RA may trigger an autoimmune response [5,6]. It is emphasized that dietary factors may have an important role in increasing the systemic inflammatory response in genetically susceptible individuals and may be an environmental factor triggering the development of RA [7]. It has been indicated that dietary factors affect inflammation, antigen presence, antioxidant defense mechanisms and intestinal microbiota, which in turn may have an impact on disease development and activity [8,9].

The “Western diet”, characterized by high fat, high protein, high sugar, excessive salt intake and consumption of processed foods, promotes obesity and may promote autoimmunity. Obesity appears to be associated with an increased risk of some autoimmune conditions, such as RA, due to a pro-inflammatory background promoted by adipocytokines [9,10]. Obesity is associated with an increased risk of developing RA in women [11]. In the systematic review by Nelson et al., it is stated that the foods consumed by RA patients affect the disease activity through direct or indirect interactions with inflammation and the immune system [12].

The objective in the treatment of patients with RA is to aim for low disease activity or remission in order to prevent structural damage and potential long-term disability [1,13]. Despite pharmacologic treatments, many patients fail to achieve the treatment goal and continue to experience pain, joint damage, functional limitation and reduced work capacity. For these reasons, additional treatment options, such as dietary interventions, are requested by patients [14].

In recent studies, the effects of foods consumed and dietary practices on the inflammatory process are more frequently discussed. In these studies, it is stated that some of the foods taken through diet have anti-inflammatory and some have pro-inflammatory properties [2,14,15]. Therefore, it has been suggested that patient-specific dietary modifications may affect the inflammation levels of patients with RA and thereby help the disease to be or remain in a remission state. However, it is recommended to support the results with further studies on this subject [16].

Therefore, in our study, we aimed to investigate the nutritional status of RA patients and the effects of the foods they consume on the patients’ inflammation, disease activity, quality of life and functional status.

## 2. Materials and Methods

This single-center cross-sectional study was performed on randomly selected adult patients who applied to the physical medicine and rehabilitation outpatient clinics for routine examination. Informed consent was obtained from all the participants at the beginning of the study, which was performed according to the Declaration of Helsinki. This study was approved by the Local Ethics Committee of Balikesir University Medical School (approval number: 2021/144). This study was also registered in the Clinicaltrials.gov database (NCT05227885).

### 2.1. Study Design and Population

This study included patients who were followed up in the physical medicine and rehabilitation clinics of Balikesir University, Health Practices and Research Hospital, between the dates December 2021 and May 2022, with a diagnosis of RA and who accepted the patient informed consent form. All the patients planned to be included in the study were diagnosed with RA by the same physical medicine and rehabilitation physician. The inclusion criteria were determined as follows: patients aged 18–75 years who met the ACR/EULAR 2010 classification criteria and were diagnosed with RA at least 2 years ago or more. We did not include patients aged 76 years and older in this study because we thought that they would have difficulty participating in the study due to additional health problems at advanced ages. The exclusion criteria were determined as follows: patients who were diagnosed with RA less than two years ago, had a change in the use of disease-modifying anti-rheumatic drugs (DMARDs) and/or drugs of the biologic agent group in their medical treatment during the last 8 weeks, use more than 10 mg of prednisolone daily, have a malignancy, are pregnant, are lactating and are unable to understand the information on the questionnaire forms.

The medical history, clinical examination, disease activity, inflammation levels and functional status evaluation of all the patients were performed by the same researcher. In addition, the body composition, anthropometric measurements, nutritional status and food consumption of all the patients were performed by the same dietician. All the study procedures were performed on the same day for each patient.

#### 2.1.1. Evaluation Parameters

##### Anthropometric Measurements

A bioimpedance device and measuring tape were used to take anthropometric measurements of the patients. The body weight, height, and waist circumference (WC), hip and upper mid-arm were measured and recorded. The body weights and compositions of individuals were measured using an MC-780 Tanita™ device (Tokyo, Japan) with a weight resistance of 150 kg (kg) and a sensitivity of 0.1 kg. The body weight was measured with thin clothing and without shoes. Their height and WC, hip and upper mid-arm were measured using a non-flexible measuring tape in accordance with the method. The height was measured with the participant standing, without shoes, feet positioned side by side, and with the patient’s head in a position where the eye and auricle were parallel. The WC was measured with reference to the anterior superior iliac crest and the lowest rib. The body mass index (BMI) was calculated using the formula obtained by dividing the weight by the square of the height.

##### Nutritional Status and Foods

In order to evaluate the nutritional status and dietary habits of the patients, an “individual food consumption record” for three consecutive days, including one day on the weekend, and a one-day “individual physical activity record” were obtained. For the individual food consumption records, patients were instructed to record their dietary intake on specified days, and one day following the completion of the records, the research dietitian reviewed and confirmed the details through questioning. The macro- and micronutrient contents of foods with a retrospective three-day nutritional record were obtained by using the BeBiS software version 9 (Ebispro for Windows, Stuttgart, Germany; Turkish Version, BeBiS 9) [17]. A retrospective 24 h “individual physical activity record form” was used to determine the physical activity status of the patients. The total energy consumption (TEC) was calculated by multiplying the time spent on activities by the physical activity ratio (PAR) determined for the basal metabolic rate (BMR) per minute. The physical activity level (PAL) was calculated by dividing the TEC by the BMR and assessed according to the WHO classification [18].

Hand grip strength (HGS) is one of the methods used to assess the nutritional status of patients and to determine the muscle function thereof [19]. The HGS of the patients was measured using a Jamar Plus Hand Dynamometer™ (Sammons Preston^®^, Bolingbrook, IL, USA). The measurements were taken with the individuals standing, ensuring full extension of the elbow and wrist. The measurements were taken twice at five-second intervals for both the dominant and non-dominant hands, and their averages were calculated and recorded in kilograms.

##### Inflammation

The systemic immune-inflammation index (SII) has been shown to measure the level of systemic inflammation in patients with RA and is a strong indicator of disease activity, joint damage and radiographic progression [20]. The SII is an index calculated using the counts of platelets, neutrophils and lymphocytes. It is determined by multiplying the platelet count by the neutrophil count and dividing it by the lymphocyte count. Higher SII values are associated with more severe RA disease activity and worse prognosis [20,21].

##### Disease Activity

The assessment of disease activity is very important in deciding on treatment and follow-up [22]. For this reason, the simple disease activity index (SDAI) and clinical disease activity index (CDAI) are used in clinical practice [13,22,23]. The SDAI is calculated as the sum of 5 variables, including the number of 28 tender and 28 swollen joints, assessment by the physician and patient of global disease activity, and serum C-reactive protein (CRP) assessment. Good reliability was found in detecting changes in disease activity [24]. The CDAI is calculated as the sum of 4 variables, including only the number of 28 tender and 28 swollen joints, assessment by the physician and patient of global disease activity, without acute phase reactants, and does not require additional tools for calculation [24,25]. Similar to the SDAI, the CDAI was initially developed for use in clinical practice and has now become reliable and valid for use in clinical trials [13]. A visual analogue scale (VAS) was used to evaluate the general health status of the patients and the global assessment by the patient and physician of disease activity. The CRP, erythrocyte sedimentation rate (ESR), platelet, neutrophil, lymphocyte values were recorded from the patient files. The number of 28 tender and 28 swollen joints, the assessment of general disease activity by the physician and the patient, and the evaluation of CRP were performed by the same physical medicine and rehabilitation specialist.

### 2.2. Sample Size

In a planned pilot study with 20 patients to determine the number of patients to be included in this study, the correlation coefficient between the nutritional values and the RA disease activity scores was found to be approximately 0.30. The sample size of the study was calculated as 92 patients, with a risk of error (α) of 5% and power (β) of 80%. Considering the 20% patient dropout rate, 110 patients were included in this study. The sample calculation was conducted using MedCalc^®^ Statistical Software version 19.7.2 (MedCalc Software Ltd., Ostend, Belgium; https://www.medcalc.org; accessed on 1 June 2021).

### 2.3. Statistical Analysis

Data analysis was performed using the statistical package program MedCalc^®^ Statistical Software version 19.7.2 (MedCalc Software Ltd., Ostend, Belgium, 2021). Descriptive statistics (mean, standard deviation, minimum, median, maximum) were used to indicate the continuous variables. The normal distribution of the continuous variables was assessed using the Shapiro–Wilk test. For continuous variables that did not follow a normal distribution, the correlation analysis was performed using the Spearman Rho correlation coefficient. Probability (*p*) values lower than α = 0.05 were considered “significant, indicating a difference between the groups”, while *p*-values higher than α = 0.05 were considered “insignificant, indicating no difference between the groups”.

## 3. Results

A total of 110 patients were included in this study, consisting of 100 (~91%) females and 10 (~9%) males. The mean age was 55.5 ± 10.3 years, the mean BMI was 30.8 ± 4.5, the mean WC was 102.5 ± 10.2 and the mean WHR was 0.65 ± 0.10 (Table 1). Table 1 presents the demographic and anthropometric data of the patients.

In our study, according to the BMI values, 20% of the patients were normal (BMI 18.5–24.9 kg/m^2^), 20% were overweight (25.0–29.9 kg/m^2^), 30% were grade 1 obese (30.0–34.9 kg/m^2^), 20% were grade 2 obese (35.0–39.9 kg/m^2^) and 10% were morbidly obese (≥40 kg/m^2^). In the health risk assessment of the patients in our study based on the WC measurements, it was found that 10% (11) of patients were at low health risk, 10% (11) of patients were at high health risk, and 80% (88) of patients were at very high health risk. At the same time, according to the WHR measurements, it was determined that ~91% (100) of patients needed nutritional therapy.

The patients who participated in this study were diagnosed with RA a mean of 7 years ago and had been receiving treatment for 5 years (Table 1). All the patients were on pharmacologic treatment, 56.4% (n = 62) in the DMARD group and 43.6% (n = 44) in the biologic agent group. Among the patients who participated in the study, ~30% (n = 32) had hypertension, ~11% (n = 12) had diabetes, and ~12% (n = 13) had both hypertension and diabetes.

No statistically significant correlation was found between the anthropometric measurements and the values of inflammation and disease activity of the patients (*p* > 0.05) (Table 2).

We observed a weak, statistically significant negative correlation between the patients’ SII values and their dietary intake of polyunsaturated fatty acids (PUFAs), omega-3, vitamin E, carotene and selenium. We observed a weak, statistically significant negative correlation between the patients’ SDAI and CDAI values and their dietary intake of total energy, total fat, omega 3, calcium, zinc, and cobalamin. Additionally, there was a weak, statistically significant positive correlation between the patients’ SDAI, CDAI, and SII values and their dietary intake of omega-6 fatty acids. We observed a weak, statistically significant positive correlation between the patients’ HGS and PAL values and their dietary intake of total energy, total fat, omega 3, omega 6, PUFAs, monounsaturated fatty acids (MUFAs), and vitamin E (*p* <0.05) (Table 3).

## 4. Discussion

In this study, we investigated the nutritional status of RA patients and the effects of the foods they consume on the patients’ inflammation and disease activity. As a result of the study, we found that the dietary fatty acids, antioxidant vitamins and minerals consumed by the patients had statistically significant effects on their inflammation and disease activity. We observed no statistically significant correlation between the anthropometric measurements and their inflammation and disease activity.

The nutritional status of patients with RA is generally not very good and most of the patients are overweight or obese [14]. BMI, WC and WHR measurements are widely used for determining obesity and body composition due to their cost-effectiveness and ease of application [26]. According to the BMI, WC and WHR, in our study, we found that 60% of the patients were obese, 80% were at very high health risk, and approximately 91% were in need of nutritional treatment.

In a study investigating the correlation between anthropometric values and disease activity in patients with RA, it was found that the BMI, body weight and WC measurements were correlated with the disease activity and CRP and ESR values, which are inflammatory markers that increased with the BMI [27]. Also, in a systematic review evaluating the effects of obesity on disease activity in RA patients, it was reported that obese patients were 40% less likely to achieve remission in RA, obesity negatively affected the disease activity and symptoms during treatment, and BMI control and obesity treatment may have positive effects on the disease course [28]. While statistically significant correlations were found between the anthropometric values and the disease activity and inflammation values in these studies, no statistically significant correlation was found in our study (*p* > 0.05) (Table 2). However, in our study, most of the patients (n = 84) were overweight or obese, and most of the disease activity scores (CDAI and SDAI values in n = 83 and n = 82 patients, respectively) were at the moderate/high disease activity level. These results show that there is a positive correlation between the BMI and the disease activity values of the patients, although not at a statistically significant level, and these results are similar to the results of other studies [27,28].

It is known that the distribution of macro- and micronutrients in the diet has different effects on inflammation [29]. It has been reported that diets rich in saturated fatty acids have pro-inflammatory effects, while PUFA have anti-inflammatory effects. It is known that dietary omega-3 fatty acids reduce inflammation by decreasing the production of inflammatory eicosanoids, adhesion molecules and cytokines, whereas a high intake of omega-6 fatty acids in contrast to low omega-3 intake increases inflammation by stimulating eicosanoid production [29,30]. The nutrients consumed by RA patients may affect the disease activity through direct or indirect interactions with inflammation and the immune system. It has been noted that dietary unsaturated fatty acids and antioxidants may reduce oxidative stress and inflammation, thereby reducing the symptoms seen in RA [12].

In the study by Tedeschi et al., it was shown that RA patients who consumed ≥2 fish per week, a rich source of omega-3 fatty acids, had lower DAS28 and CRP scores compared to RA patients who consumed no fish or less than 1 fish per month [31]. Studies have reported that diets rich in omega-3 PUFA reduce inflammation and suppress disease activity in RA [29,30,31]. In a systematic review, it was reported that omega-3 fatty acid intake decreased the duration of morning stiffness and the number of tender and swollen joints in RA patients, suggesting that omega-3 fatty acids may have a therapeutic role [30]. In our study, we found that especially omega-3 fatty acids, one of the fatty acids taken with nutrition, had positive effects on the inflammation level and disease activity of the patients. In our study, omega-3 fatty acids reduced the inflammation and disease activity in patients with RA through anti-inflammatory effects, which is consistent with the results of other studies.

Many studies have reported that certain dietary vitamins and antioxidant micronutrients have an important role in the prevention and/or reduction of oxidative stress caused by reactive oxygen species in the inflammatory process [32]. Antioxidants such as vitamin E, carotenoids, vitamin C, selenium, and zinc have been reported to have positive effects on inflammation [33]. However, it has been noted that increased consumption of refined grains with a high glycemic index, trans and saturated fatty acids may have a pro-inflammatory effect [34,35]. When the effects of dietary nutrients on the disease in RA are investigated, it has been reported that elements such as omega-3 fatty acids, vitamins, oleic acid in olive oil contained in the Mediterranean diet may have positive effects on disease activity and progression with the help of their anti-inflammatory properties [36]. In a randomized controlled trial investigating diet and disease activity in RA patients, an anti-inflammatory dietary model with higher intakes of PUFA, eicosapentaenoic acid, decosapentaenoic acid, vitamin E, and selenium and lower intakes of saturated fat was found to suppress disease activity [15]. In our study, we found that vitamin E, cobalamin, carotene, Se and Zn intake in the diet had positive effects on the inflammation and disease activity of the patients. Consistent with the results of previous studies, we found that dietary intake of anti-inflammatory PUFA, omega-3, vitamin E, carotene and selenium decreased inflammation, whereas pro-inflammatory omega-6 fatty acids increased inflammation.

In recent years, the effects of dietary intake and dietary practices on the inflammatory process have been mentioned more frequently and the number of studies on this subject has increased. While most of the previous studies used the CRP or ESR values to evaluate the inflammation level in patients with RA, we used the SII to assess the level of inflammation in patients with RA, which has been validated and shown to be reliable and a strong indicator of disease activity, joint damage and radiographic progression [20]. Furthermore, instead of the DAS28, which has been used in many studies to assess disease activity in RA, we used the CDAI and SDAI, which show a good correlation with the DAS28 and whose reliability and validity have been established for use in clinical studies [13].

As a result of this study, we found that dietary fatty acids, antioxidant vitamins and minerals had significant effects on the inflammation and disease activity of the patients. Similar to the results of other studies, the data obtained from the results of our study suggest that the addition of nutritional therapies and recommendations to the pharmacologic treatments of patients with RA may help patients to maintain low disease activity or achieve remission.

### Limitations

This trial also has some limitations. The disease is more common among women than men and this is mirrored in our study sample, where men make up a minority. Patients continue their medical treatments, which can affect nutrition, inflammation, and disease activity.

## 5. Conclusions

The data obtained from this study suggest that, in addition to the current pharmacological treatments, regulating the nutrition of RA patients with adequate, balanced, anti-inflammatory diet programs and recommendations may help to reduce inflammation levels, keep disease activity at a low level or achieve remission. We think that the findings will provide valuable information to healthcare professionals, allowing them to design comprehensive and personalized nutritional approaches to improve the well-being of RA patients. However, these suggestions should be supported by randomized controlled studies.

## Figures and Tables

**Table 1:** Baseline characteristics of patients with rheumatoid arthritis.

Variables		Results	
		Mean ± SD or Number (%)	Median (Min–Max)
Demographic			
Age		55.5 ± 10.3	
Gender	Male	10 (%9.09)	
	Female	100 (%90.91)	
Anthropometry			
BMI, kg/cm^2^		31.6 ± 6.5	
Waist circumference, cm		102.5 ± 10.2	
Waist/height ratio		0.65 ± 0.10	
Hip circumference, cm		111.6 ± 11.6	
Total body fat, kg		28.5 ± 10.4	
Total body water, kg		34.9 ± 5.6	
Lean body mass, kg		49.2 ± 7.9	
RA-related			
Durations of diseases (year)			7 (2–30)
Durations of therapy (year)			5 (2–30)
CDAI		25.1 ± 14.2	
SDAI		27.2 ± 15.3	
SII		1068.1 ± 4345.9	
HAQ		0.71 ± 0.57	
Physical activity			
HGS		18.9 ± 6.9	
PAL		1.5 ± 0.1	

Variables are expressed as mean ± SD, frequency (%) or median (interquartile range). BMI: body mass index; CDAI: clinical disease activity index; SDAI: simple disease activity index; SII: systemic immune-inflammation index; HAQ: health assessment questionnaire; HGS: hand grip strength; PAL: physical activity level; kg: kilogram; cm: centimeter.

**Table 2:** Correlations between the parameters of disease activity, systemic immune-inflammation index, functional conditions and anthropometric values in patients with rheumatoid arthritis.

r/p	CDAI	SDAI	SII	HAQ
BMI	0.003/0.98	−0.005/0.959	−0.095/0.345	0.063/0.531
TBF	−0.019/0.853	−0.018/0.857	−0.07/0.489	0.069/0.495
Hip circumference	−0.015/0.884	−0.017/0.868	−0.112/0.264	0.033/0.746
Waist circumference	−0.046/0.644	−0.027/0.79	−0.018/0.861	0.062/0.536
Waist/height ratio	0.007/0.943	0.023/0.818	−0.056/0.58	0.136/0.176

BMI: body mass index; TBF: total body fat; CDAI: clinical disease activity index; SDAI: simple disease activity index; SII: systemic immune-inflammation index; HAQ: health assessment questionnaire; r: correlation coefficient; *p* < 0.05 statistically significant.

**Table 3:** Correlations between the parameters of disease activity, systemic immune-inflammation index, hand grip strength, physical activity level and nutritional status values in patients with rheumatoid arthritis.

r/p	CDAI	SDAI	SII	HGS	PAL
Energy (kcal)	**−0.19/0.04**	**−0.21/0.03**	0.039/0.7	**0.20/0.03**	**0.20/0.04**
Protein (g)	−0.12/0.20	−0.17/0.07	−0.09/0.36	0.16/0.09	0.15/0.11
Carbohydrate (g)	−0.08/0.39	−0.10/0.28	0.10/0.27	0.17/0.08	0.11/0.24
Oil (g)	**−0.19/0.04**	**−0.20/0.04**	0.03/0.76	**0.18/0.04**	**0.19/0.04**
Fiber (g)	0.00/0.97	−0.05/0.57	−0.01/0.86	0.06/0.51	0.17/0.08
Saturated oil	−0.09/0.32	−0.10/0.30	0.04/0.64	0.01/0.85	0.06/0.55
MUFAs (g)	−0.15/0.11	−0.18/0.06	−0.13/0.18	**0.23/0.21**	**0.17/0.04**
PUFAs (g)	−0.08/0.41	−0.07/0.46	**−0.22/0.02**	**0.27/0.00**	**0.29/0.00**
Omega 3 (g)	**−0.20/0.04**	**−0.18/0.04**	**−0.21/0.02**	**0.22/0.02**	**0.21/0.02**
Omega 6 (g)	**0.19/0.04**	**0.18/0.03**	**0.20/0.03**	**−0.28/0.00**	**−0.31/0.00**
Carotene (mg)	0.05/0.57	0.00/0.93	**−0.20/0.04**	0.13/0.17	0.14/0.14
Vit. B6 (mg)	−0.07/0.47	−0.13/0.19	−0.09/0.34	−0.04/0.65	−0.00/0.98
Vit. B12 (µg)	**−0.22/0.02**	**−0.26/0.00**	−0.08/0.38	0.08/0.39	0.3/0.76
Vit. C (mg)	0.053/0.6	−0.01/0.89	−0.00/0.93	0.05/0.58	0.07/0.48
Vit. E (mg)	−0.09/0.32	−0.11/0.24	**−0.19/0.04**	**0.33/<0.001**	**0.33/<0.001**
Calcium (mg)	**−0.21/0.02**	**−0.22/0.02**	−0.01/0.91	0.14/0.14	0.14/0.08
Zinc (mg)	**−0.18/0.04**	**−0.24/0.01**	−0.12/0.21	0.75/0.45	0.10/0.29
Selenium (µg)	0.08/0.41	0.08/0.40	**−0.19/0.04**	−0.00/0.93	−0.12/0.21

CDAI: clinical disease activity index; SDAI: simple disease activity index; SII: systemic immune-inflammation index; HGS: hand grip strength; PAL: physical activity level; MUFAs: monounsaturated fatty acids; PUFAs: polyunsaturated fatty acids; Vit: vitamin; g: gram; mg: milligram; µg: microgram; kcal: kilo calories; r: correlation coefficient; *p* < 0.05 statistically significant.

## Data Availability

The data that support the findings of this study are available from the corresponding author upon reasonable request.
